# RETRACTION: The SP/NK1R System‐Mediated ROS Generation in GBM Cells through Inhibiting Glutaredoxin Protein

**DOI:** 10.1155/nri/9814360

**Published:** 2026-09-16

**Authors:** Neurology Research International

RETRACTION: N. Mehrabani, M. R. Vaezi Kakhki, H. Javid, S. Ebrahimi, and S. I. Hashemy, “The SP/NK1R System‐Mediated ROS Generation in GBM Cells through Inhibiting Glutaredoxin Protein,” *Neurology Research International* 2021, no. 1 (2021): 9966000, https://doi.org/10.1155/2021/9966000.

The above article, published online on 07 December 2021 in Wiley Online Library (https://wileyonlinelibrary.com), has been retracted by agreement between the journal Associate Editor, Richa Sharma and John Wiley & Sons Ltd. The retraction has been agreed after an investigation into data concerns raised by *Coleophora glitzella* via PubPeer [1]. Specifically, that the cell viability curve in Figure 1 does not exhibit a typical sigmoidal dose‐response curve, and lacks errors bars, despite the authors claiming to have run three replicate experiments.

In addition, the journal investigated data concerns raised by a Reader. Specifically, Figure 1 shares unexpected similarities with several figures published across other articles which share some authors [2–4]. The ROS data in Figure 2 also share unexpected similarities with several figures published across other articles which share some authors [5–11].

The authors remained unresponsive to the journal’s attempts to contact them. As such, the Editor cannot consider the results to be reliable. This article is therefore retracted.
